# App‐Supported Assessment of Clinical Courses for Dental Students: Discrepancies between Self‐Assessment and Instructor Assessment

**DOI:** 10.1002/jdd.13938

**Published:** 2025-05-13

**Authors:** Janosch Goob, Anja Liebermann, Oliver Schubert, Isabel Lente, Kurt Erdelt

**Affiliations:** ^1^ Department of Prosthetic Dentistry University Hospital LMU Munich Munich Germany; ^2^ Department of Prosthetic Dentistry Faculty of Medicine and University Hospital Cologne University of Cologne Cologne Germany

**Keywords:** assessment | instructor assessment | self‐assessment | self‐evaluation | teaching assessment

## Abstract

**Introduction:**

From a student's perspective, assessment plays a central role in education and is essential in a university setting. Developing accurate self‐assessment, without over‐ or underestimating one's abilities, requires practice and is often misaligned with actual skills. This study examines the gap between student self‐assessments and instructors' assessments in everyday dental courses, focusing on overestimation, underestimation, and accurate self‐assessment.

**Methods:**

The app “digital course organizer” for organization and assessment was used to compare self‐assessment and instructor evaluation (student and teacher) for each day of patient treatment at a university hospital. Data were collected over four semesters from 309 students resulting in a total of 15312 dual assessments. These were analyzed for tendencies toward overestimation, underestimation, or neutral assessment. Discrepancies between student self‐assessments and evaluations by instructors were examined across four key criteria i) quality of treatment; ii) support from the teaching doctor; iii) theoretical knowledge and iv) professional appearance and organization.

**Results:**

The statistical results across all assessments showed a predominantly neutral difference between the assessment outcomes of students and instructors. Further statistical analysis of the differences in assessment results between clinical courses showed no significant differences (*p* ≥ 0.128).

**Conclusions:**

The results demonstrated a predominantly neutral correlation between students' self‐assessments and the assessments provided by instructors in dental clinical courses. The findings indicate that students' self‐assessments were largely aligned with those of the instructors, showing no significant discrepancies between student expectations and instructor assessment.

## 1 Introduction

In clinical professional teaching, the focus goes beyond the acquisition of sheer knowledge or practical skills to include the important aspect of accurate self‐assessment through critical evaluation [1]. This is an essential tool for promoting and improving the clinical performance of future practitioners [2]. Education is largely driven by assessment which is an essential part of higher education [1]. It is a process of measuring students' knowledge, behavior, development, and skills, with the aim of facilitating learning and providing learners with individually tailored information [1, 3]. Effective feedback in this context is specific, and timely, encourages self‐assessment, and leads to the achievement of learning objectives [4].

Assessment formats can be classified as summative (assessment of learning) or formative (assessment for learning). In recent times, formative assessment has become increasingly important [5, 6, 7]. Summative assessment serves as a conclusive summary of achievement, typically at the end of a process, and assigns a final grade [6]. A drawback is that positive or negative development is exclusively assessed at the end of a process and focuses on an existing outcome. Formative assessment plays a crucial role in supporting students' academic development by facilitating timely interventions to address potential shortcomings [4, 8]. Unlike summative assessments, it offers continuous feedback, which helps identify discrepancies between students' expectations and actual performance [9]. Beyond that, the continuity of assessment provides guidance, reduces uncertainty, facilitates more focused and efficient growth in skills and knowledge [10], and has a positive impact on learning and performance [11]. Assessment should be seen as a structured and continuous process that provides a comprehensive picture over time, rather than just a series of individual evaluations leading to a grade [5, 6].

In addition to the two main types of assessment, namely summative and formative assessment, it is also possible to divide assessment into two further categories: self‐assessment and instructor assessment. Self‐assessment in a university setting presents several advantages. It encourages self‐reflection and critical thinking by enabling students to evaluate their own progress and performance. Students are encouraged to engage in critical reflection on their strengths and weaknesses and to consider how they might improve. For healthcare providers in particular, the acquisition of self‐assessment is an essential element in the promotion of professional competence, professional morale, and the skills associated with self‐directed, lifelong learning [12, 13, 14]. When students take personal responsibility for evaluating their own understanding during the educational process, they become part of the assessment process and encourage their self‐management of their learning [15]. Students may utilize self‐assessment as a means of monitoring their own progress and identifying areas for improvement, thereby enabling them to identify deficiencies in their competence [15]. On the other hand, the assessment provided by the instructors offers an expert perspective. Instructors have extensive knowledge and experience in their field and their assessment can provide valuable insights into a student's progress. Instructor assessment can provide more accurate and reliable assessments and might probably provide a fair judgment. Teachers who are closely involved in the assessment process can use the results to enhance their teaching and support strategies, ensuring that they are tailored to the individual needs of their students.

Previous research has already shown that low‐performing students paradoxically tend to overestimate their performance or knowledge [12, 13, 14, 15, 16, 17]. Whereas high‐achieving students are more cautious and even harsh in their self‐assessment, with a tendency to underestimate [14, 15, 16, 17]. This empirical evidence of meta‐ignorance is described by the “Dunning‐Kruger Effect”, which postulates that students who lack competence are also likely to be unable to assess their own competence—or non‐competence [18, 19, 20].

This investigation aimed to evaluate the accuracy of self‐ and instructor‐assessment of academic performance in day‐to‐day clinical dental courses in terms of overestimation, underestimation, and neutral assessment. An app is used to provide students with a daily dual assessment (self‐assessment and instructor assessment) of the individual work sequences of each treatment day in clinical dental courses.

The following hypotheses were tested: (1) there is no significant difference between self‐assessment and instructor assessment, and (2) there is no improvement in self‐assessment over time.

## 2 Methods

The study was approved by the Ethics Committee of the Medical School (Project No. 21‐0313) and declared unobjectionable.

The investigation was a survey‐based dual‐assessment study among dental students from the clinical study phase of the Department of Prosthetic Dentistry at a German university hospital over a period of four semesters. The eighth and 10th semesters (fourth and fifth year) are devoted to prosthetic dentistry with an emphasis on fixed and removable dental prostheses. Dental students from the eighth and 10th semesters treat patients with prosthetic treatment needs under the supervision of a professional instructor (graduated dentist who practices the profession and covers a teaching role at dental college) and senior practitioner (experienced dentist who manages the clinical courses and supervises all clinical activity). Each semester was supervised by four instructors, with each instructor accompanying the same group of students for the duration of the semester.

The app “Digital Course Organizer” (DCO) is designed to help students simplify and digitize their organization in clinical courses. Furthermore, the DCO is equipped with a dual assessment tool for self‐assessment by students and expert assessment by instructors after daily dental clinical courses. Instructors and students were familiarized with the DCO and the assessment items at the beginning of the semester and were calibrated by the supervising senior practitioners. After each day of treatment, students self‐assessed their own performance via the DCO app based on the following criteria: i) quality of treatment; ii) support from the instructors; iii) theoretical knowledge, and iv) professional appearance and organization. The criteria, questions, and evaluation tools (Visual Analog Scale [VAS]) for the assessment had been developed by a professional team within the Department of Prosthetic Dentistry.

### 2.1 Students’ Self‐assessment

Each student had a private login for the app to complete the survey anonymously. Other students had no access to this personalized self‐assessment. Only the student's instructor could access it and provide an instructor assessment. After each treatment session, students were required to self‐assess the treatment steps based on the assessment criteria within 24 h. The assessment process was linked to each student, the type of work, the individual treatment steps, and the specific day of treatment. The VAS for the different assessment criteria ranged from 1 to 10 (0%–100%) and was marked by the students using a scroll bar on the line. Within 24 h after patient treatment, students completed their self‐assessment before receiving any assessment from the instructors to avoid bias.

### 2.2 Instructors´ Expert‐assessment

**FIGURE 1:**
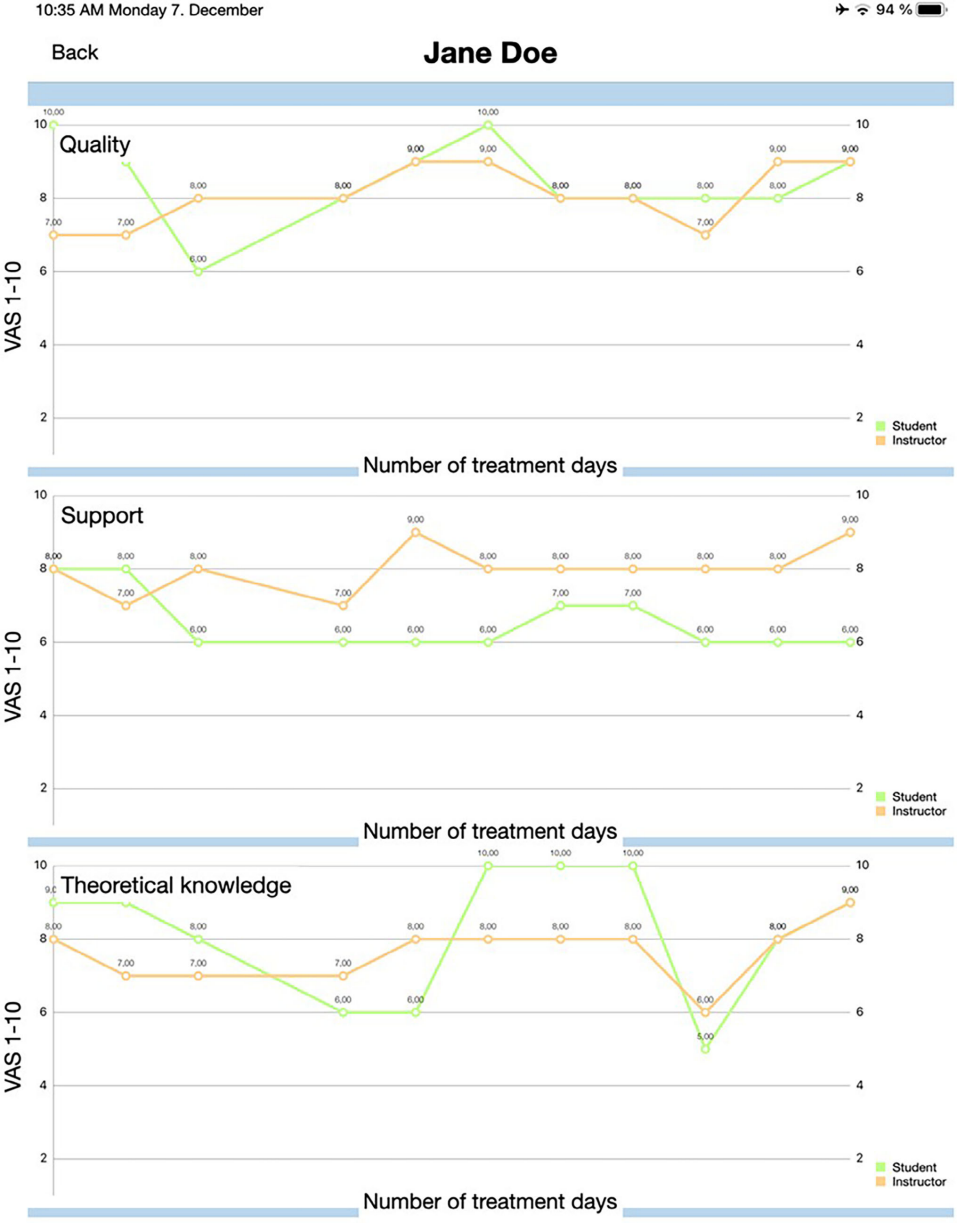
Graphical illustration of self‐assessment (green) and instructor assessment (orange) for each day of treatment. Differences between self‐assessment and instructor assessment reflect overestimation, underestimation, and neutral assessment.

Since students completed the self‐assessments, instructors assessed each student using the same criteria without knowing the outcome of the students' self‐assessments to avoid bias. Likewise, instructors were required to complete the assessment within 24 h. Merging the data enabled the graphical visualization of the discrepancy between self‐ and instructor assessment in the app for each student, each treatment, and each day (Figure 1). All students were encouraged to critically analyze the discrepancies between their self‐assessment and that of their instructors in order to evaluate the accuracy of their self‐assessment. In cases of unresolved issues or significant discrepancies in the assessments, students were encouraged to engage in a constructive dialogue with their instructors to gain a deeper understanding of the underlying issues.

The dual‐assessment data were collected over four semesters with a total of 309 students and 15,312 assessments. All students (100%) used the app to plan and document their daily treatment routines and to evaluate the individual treatment steps. The data were analyzed in terms of overestimation, underestimation, and neutral assessment and according to the following aspects:

1. The total data set without subdivision by semester and assessment criteria.
2. The data set for each semester (eighth and 10th) without subdivision by assessment criteria.
3. The total data set with subdivision by assessment criteria.
4. The data set for each semester (eighth and 10th) with subdivision by assessment criteria.

### 2.3 Statistical Analysis

Data were analyzed using the statistical program SPSS 26.0 (IBM, New York, NY, USA). For sample size calculation regarding the number of students and assessments, the G*Power (G*Power version 3.1.9.7; HHU Düsseldorf, Germany) analysis was used. First, the difference scores between the students' and teaching doctors' assessments were calculated. The normal distribution was tested using the Kolmogorov‐Smirnov test. Second, non‐parametric analysis was performed using the Wilcoxon test for paired samples to determine whether the median of the difference between the students' and teaching doctors' assessments showed a significant deviation from 0. If the difference was not significantly different from 0, the assessment was considered neutral. If it was significantly different, the median of the difference was used to distinguish between underestimation and overestimation. This procedure allowed for the assignment of the students to one of three evaluation groups for the study period. These assessment groups were: underestimation, neutral, or overestimation. These groups were combined and analyzed using cross tables. Using the chi‐square test and the chi‐square post hoc test with Bonferroni correction, the cross tables were analyzed for their significant dependencies. The significance level was set at *p* ≤ 0.05.

## 3 Results

Seventy percent of the assessment difference values showed a deviation from the normal distribution, resulting in the use of non‐parametric tests. Based on the G*Power analysis, a sample size of 172 students and seven assessments per student were estimated as necessary to achieve a meaningful result. The overall results of the assessments of both clinical courses across all semesters are depicted in Table 1. The statistical results from the cross table showed a predominantly neutral difference between the assessments of students and teaching doctors (Table 1). Further statistical analysis (Chi‐quadrat test) of the differences in assessment results between both clinical courses showed no significant differences (*p* ≥ 0.128). The separate assessment results for both clinical courses are presented in Table 1. Analyzing the assessment criteria, applying the Chi‐square post hoc test with Bonferroni correction, a significant difference from the expected value for overestimating (*p* = 0.012) and underestimating (*p* < 0.001) was observed in the eighth semester for the assessment criterion “help from instructor” in student evaluations (Table 2). In the 10th semester, a significant difference was found for overestimation (*p* = 0.002) for the same assessment (Table 2). All other scores for the different assessment criteria showed no significant differences from the expected value (*p* ≥ 0.149). Detailed assessment results for the different criteria are presented in Table 2.

**TABLE 1:** Assessment of clinical courses, in total and separately.

	Neutral (%)	Overestimation (%)	Underestimation (%)
**Clinical course 1 and 2 (total data set)**	43.9	30.4	25.7
**Clinical course 1 (8th)**	43.7	31.5	24.8
**Clinical course 2 (10th)**	44.1	29.4	26.5

**TABLE 2:** Assessment of the evaluation criteria in total and separately.

	Neutral (%)	Overestimation (%)	Underestimation (%)
**Assessment criteria in total (courses 1 and 2; 8th and 10th semester)**			
**Quality of treatment**	39.6	39.3	21.1
**Support from the instructor**	45.2	15.2	39.6
**Theoretical knowledge preparation**	48.0	30.3	21.8
**Professional appearance/organization**	42.9	36.7	20.4
**Assessment criteria clinical course 1 (8th semester)**			
**Quality of treatment**	43.2	38.7	18.1
**Support from the instructor**	39.4	**16.8**	**43.9**
**Theoretical knowledge preparation**	48.7	31.2	20.1
**Professional appearance/organization**	43.6	39.1	17.3
**Assessment criteria clinical course 2 (10th semester)**			
**Quality of treatment**	36.3	39.9	23.8
**Support from the instructor**	50.6	**13.7**	35.7
**Theoretical knowledge preparation**	47.2	29.5	23.3
**Professional appearance/organization**	42.3	34.5	23.2

Significant differences are marked in bold type.

## 4 Discussion

The present study examined the discrepancy between students' self‐assessments and those of professional instructors with regard to four key criteria: i. quality of treatment; ii. support from the instructor; iii. theoretical knowledge preparation, and iv. professional appearance and organization, and analyzed the difference in terms of overestimation, underestimation, and neutral assessment.

The first hypothesis, which posited that there is no significant discrepancy between self‐assessment and assessment by instructors, can be partially accepted. The results indicated a neutral difference between the assessment outcomes (student/instructor) across both courses and all values. Self‐ and instructor‐assessments matched in 43.9% of cases, 30.4% overestimated themselves, and 25.7% underestimated themselves. Overall, this investigation suggests no prevalent tendency for either overestimation or underestimation among dental students regarding the assessment criteria. However, a significant difference was observed for the assessment criterion “help from the instructor”. Upon analyzing the individual assessment criteria, a significant deviation from the expected value for overestimation and underestimation was identified in the eighth semester concerning the assessment criterion “help from the instructor”. This indicates that students in the eighth semester may have difficulty accurately assessing the level of assistance required from the instructing staff. Students tend to overestimate or underestimate the level of help needed, leading to inaccurate assessments. In contrast, for the 10th semester, only a significant difference in overestimation by students was identified for the assessment criterion “help from the instructor”. Consequently, some students may have accepted or required more assistance than they anticipated. Students in their 10th semester are likely to have more experience than their counterparts in their eighth semester, having already treated patients in prosthodontic courses. They have acquired practical knowledge under the guidance of experienced dentists, which may lead to fewer difficulties in assessing patients, greater confidence in patient interactions, and higher proficiency due to their familiarity with the procedures involved. All other scores for the various assessment criteria showed no significant deviations from the expected values. The second hypothesis, suggesting no improvement in self‐assessment over time, is accepted, except for the assessment criterion “help from the instructor”. As discussed earlier, experienced students in the 10th semester demonstrated a better ability to evaluate the help provided by instructors. Students in the 10th semester have more clinical experience and are more confident in their expertise. However, the overall results regarding the improvement of assessment over time revealed a predominantly neutral difference between both semesters. The differences in assessment results between both semesters showed no significant differences (*p* ≥ 0.128). Therefore, it can be inferred that there has been no significant improvement in the ability to self‐assess over time, i.e. from the eighth to the 10th semester.

It is notable that, in general, there is no discernible inclination toward either overestimation or underestimation. A review of the literature on self‐assessment and evaluation reveals that students often exhibit a tendency to overestimate their abilities [12, 13, 14, 15, 16, 17]. Another significant observation in literature is the Dunning‐Kruger effect, which posits that individuals who perform poorly in a particular domain are also the least accurate in assessing their own abilities [4]. Self‐assessment is essential for healthcare professionals to accurately identify their skills, knowledge, weaknesses, and strengths, promoting lifelong learning. Identifying errors in treatment has the potential to enhance competence and quality of work, benefiting both students and experienced practitioners. Several studies have demonstrated the efficacy of self‐assessment as an educational tool, with students recognizing its value [21, 22]. Furthermore, self‐assessment has been shown to enhance student confidence in their performance [23].

The findings indicate that a considerable number of students in clinical courses do not fit the pattern of overestimation or underestimation. This does not necessarily imply an accurate self‐assessment but rather suggests a lack of a tendency to overestimate or underestimate compared to the assessment by expert instructors.

### 4.1 Strengths, Limitations, and Future Research

One strength of the study lies in the large number of evaluations conducted over a four‐semester period. However, it is important to note that the data represent the results of one specific university and cannot be generalized. The diversity of teaching methods and approaches across universities makes it difficult to draw universal conclusions on this topic. The 24‐h evaluation time frame may have contradictory effects on the assessment. Immediately after treatment, students´ self‐reflection can be biased by emotional factors, whereas an evaluation after 24 h may also lead to distorted results. A study examining whether the elapsed time between teaching and student evaluations affects the quality ratings in preclinical courses reached the following conclusion: The time between the event and its evaluation has only a negligible impact on the final evaluation outcome [24]. Nevertheless, the evaluation of dental procedures performed on a patient for the first time presents a challenge for self‐assessment. In addition, the supervising instructor also influences the outcome, as each instructor has a different level of knowledge and practice experience. A highly knowledgeable assessor is more likely to provide accurate and fair assessments [25]. It is possible that interpersonal relationships may influence the results. Despite these limitations, the results are useful for further investigations. Conducting a multicenter investigation with dual assessment across different universities could provide more data, hence more clarity, and a better understanding of this topic. Further research should formulate more specific and clearer questions, as those in this study are rather general and open to interpretation.

## 5 Conclusion

The findings suggest a general alignment between students' self‐assessments and the evaluations provided by instructors in dental clinical courses. However, a substantial proportion of assessments exhibited discrepancies, although only a limited number of these differences reached statistical significance. This implies that students' self‐assessment of their performance was closely aligned with the expert assessment, with no significant overall discrepancies.

From a didactic perspective, the dual assessment provides a didactic basis for the professional instructor to objectively evaluate the students' current level of performance at the relevant time, thus enabling the provision of appropriate guidance. For the students, the assessment by the instructors provides valuable feedback that can contribute to their ongoing learning. This feedback can also help them to accurately assess their own performance.

## Conflicts of Interest

The authors declare no conflict of interest.

## Funding information

The research leading to these results has not received any funding.

## Data Availability

The data will be provided on request.
